# Dietary patterns of Indian school‐aged children and associations with markers of chronic disease risk

**DOI:** 10.1002/fsn3.3631

**Published:** 2023-08-16

**Authors:** Geeta Trilok‐Kumar, Anku Malik, Yamini Gusain, Eve Millerot, Renuka Pathak, Suzanne Filteau

**Affiliations:** ^1^ Institute of Home Economics, University of Delhi New Delhi India; ^2^ London School of Hygiene and Tropical Medicine London UK

**Keywords:** body composition, child nutrition, children, chronic disease, dietary pattern, India, public health

## Abstract

There is an increasing burden of noncommunicable diseases (NCDs) in India which may be related to changing dietary patterns. We aimed to assess dietary patterns in children since they have time to change unhealthy patterns before NCDs develop. Participants were 665 children, 9–12 years old, born with low birth weight and 77 similarly aged normal birth weight controls. We collected data on sociodemography, anthropometry, body composition, and markers of risk for NCDs: grip strength, long jump, hemoglobin A1c (HbA1c). A food frequency questionnaire was used to collect dietary data from which dietary patterns were derived using principal component analysis (PCA). Fourteen food groups were included in the PCA analysis, resulting in three components: ‘fruits and vegetables’, ‘protein’, and ‘sugar and fat’. Higher socioeconomic status and maternal education were associated with lower adherence to the fruit and vegetable pattern and higher adherence to the protein and sugar and fat patterns. Adherence to the fruits and vegetables pattern was associated with lower height‐for‐age, whereas the fat and sugar pattern was associated with higher indicators of body fat. In linear regression analyses adjusted for age, sex, religion, socioeconomic status, maternal education, and season of data collection, adherence to the ‘fruits and vegetables’ pattern was associated with lower grip strength, shorter long jump, and lower HbA1c. Adherence to the other patterns was not associated with NCD risk factors. Higher consumption of fruits and vegetables, achievable even by poorer families in the cohort, may lower HbA1c, a risk factor for diabetes.

## Graphical abstract

There is an increasing burden of noncommunicable diseases in India related to changing dietary patterns. We assessed dietary patterns in children. Adherence to the fruits and vegetables pattern was associated with lower HAZ, whereas the fat and sugar pattern was associated with higher indicators of body fat. Higher consumption of fruits and vegetables may lower HbA1c, a risk factor for diabetes.

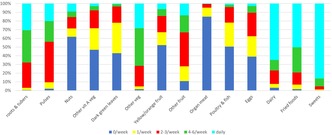

## 1 INTRODUCTION

The nutrition transition is evident in India where 18% of children are born with low birth weight (International Institute for Population Sciences and ICF, 2017), 17% of children under 5 years are wasted, and 35% are stunted (International Institute for Population Sciences and ICF, 2017), while the prevalence of overweight and obesity among school‐aged children and adolescents is 4%, and over 20% among adults (International Institute for Population Sciences (IIPS) and ICF, 2020). Urbanization and changes to the physical and social environments play a major role in increasing the double burden of under‐ and overnutrition (Popkin et al., 2020). In addition, there is evidence of a life course contribution whereby undernutrition in early life, if followed by access to large amounts of calorie‐dense food later in life, may increase the risk of noncommunicable diseases (NCDs) associated with overweight, as suggested by the capacity‐load model (Wells, 2010).

Overweight and its consequences are driven by a combination of excess calories and inadequate amounts of exercise (World Health Organization, 2021). Urban children, other than those from high‐income families, may lack opportunities to exercise due to limited playgrounds and streets unsafe for play due to traffic. Ameliorating this situation would require major changes to urban infrastructure. These same children may have ready access to nutrient‐poor and energy‐dense foods. Our previous work in Kathmandu, Nepal showed that even children under 2 years had very high intakes of commercial energy‐dense foods which were associated with stunting and micronutrient deficiencies (Pries et al., 2019). Intake of such foods may increase as children get older and have greater access to foods outside the home. Understanding the diets of urban children from low‐income families will be important for designing interventions to reduce the impact of poor diet on later NCDs.

Overall dietary patterns appear to be more closely associated with overweight and its consequences than individual nutrients or foods (Hoffmann et al., 2004; Garcia‐Chavez et al., 2020; Zhao et al., 2021). There are multiple approaches which can be used to reduce large dietary intake databases to a few patterns (Zhao et al., 2021). One commonly used approach which provides information that can readily be used to design diet interventions is principal component analysis (PCA; Manyanga et al., 2017; Richter et al., 2012). However, PCA generates intake patterns based solely on the diet data and these descriptive patterns will not always be associated with the health outcomes of interest, e.g., overweight or NCD risk.

We aimed to use PCA to derive dietary patterns and their associations with risk factors for NCDs among a group of urban Indian children who are at high risk for adult NCDs because most were born with low birth weight but are growing up in an obesogenic environment with high access to energy‐dense foods but limited access to play areas or exercise. We wished to determine whether particular dietary patterns were associated with NCDs in order to plan future research on health and diet interventions in this population.

## 2 METHODS

### 2.1 Design and participants

The study, conducted from 2019 to 2021, combined a follow‐up of an existing cohort of children born at term with low birth weight (<2.5 kg; Trilok‐Kumar et al., 2011) with a cross‐sectional study of newly recruited control children born with documented normal birth weight and belonging to the neighborhood areas of the DIVIDS cohort in the urban slums of Delhi. The cohort was originally recruited as neonates in 2007–2010 for the Delhi Infant Vitamin D Supplementation (DIVIDS) trial (Trilok‐Kumar et al., 2011) and was previously followed up when aged about 5 years (Trilok‐Kumar et al., 2015). The dietary data were collected as part of the third follow‐up of the DIVIDS cohort to understand the effects of being born LBW on later NCDs. For eligibility, NBW children needed to be of a similar age to DIVIDS children and to have documented, on their health card, birth weight of >2.7 kg. This was to ensure their birth weight was different from that of the DIVIDS children, even accounting for errors in routine clinic weight measurements. It was difficult to recruit controls since many families of children this age no longer had birth weight documents so the birth date range for control children was extended to 2012.

### 2.2 Sociodemography, anthropometry, body composition, and markers of risk for chronic diseases

Children came with at least one parent or guardian to the Institute for Home Economics, Delhi University. After taking informed consent from the parents/guardian and assent from the children, sociodemographic data were collected by questionnaire. Anthropometric measurements were taken in triplicate using standard methods (Gibson, 2005) and the median was used in analyses; data for weight, height, waist, hip, and mid‐upper arm circumferences, triceps, and subscapular skinfolds were collected. Body fat and fat‐free mass were measured by bioelectrical impedance using a Tanita MC980MA, Tokyo, Japan.

Grip strength was measured three times with each hand and the maximum value was used in analyses. As a similar measure of strength for legs, children were asked to jump as far as possible and the greatest distance of three attempts was used in analyses. Hemoglobin A1C (HbA1c) was measured in venous blood collected and analyzed at Lal laboratories, Delhi.

### 2.3 Diet data collection

A qualitative Food Frequency Questionnaire (FFQ) was used to collect data on foods consumed by the children in the past week. We generated 16 food categories, based on their common characteristics and uses and on data from our previous study on children's dietary intakes using weighed food records (unpublished). Estimated frequency of intake over the past week was recoded as <1/week, i.e., almost never, 1 time/week, 2–3 times/week, 4–6 times/week, and daily. Two food groups—grains and cooking oil plus butter or ghee—were eaten daily by over 97% of the population so would not have been useful for distinguishing different diet patterns and were, therefore, dropped from diet pattern analyses. The 14 food groups included in the diet pattern analyses were, therefore: (1) roots and tubers, (2) pulses, (3) nuts, (4) vitamin A‐rich vegetables not including dark green leafy vegetables (DGLV), (5) DGLV, (6) other vegetables, (7) orange and yellow fruits, (8) other fruits, (9) organ meat plus red meat (since they are cooked and eaten together as a curry), (10) poultry or fish, (11) eggs, (12) dairy, i.e., milk, yogurt, cheese or other milk products, (13) fried savory foods, and (14) sweets including cake, biscuits, and candy.

### 2.4 Data preparation for statistical analyses

Statistical analyses were conducted using Stata 17. Body mass index (BMI) and fat and fat‐free mass indices (FMI and FFMI) were calculated as kg/height^2^. *Z* scores for height (HAZ) and BMI (BMIZ) were determined using the World Health Organization standards in the Stata zanthro command. FMI and anthropometric variables related to fat—BMI, waist circumference, triceps, and subscapular skinfolds—were log‐transformed in order to normalize distributions; medians and 25th and 75th percentiles are presented in descriptive data. Socioeconomic status (SES) was determined using a PCA of family and household characteristics (mother's marital status, source of drinking water, type of toilet, renting or owning their home, house quality) and family ownership of assets (mattress, car, bicycle, motorcycle, refrigerator, television, clock, radio, washing machine, air conditioner, microwave, sewing machine, computer, private internet connection). The first component was divided into terciles for analysis. We anticipated that the season of data collection could affect both diet (because of both food availability and festivals) and NCD outcomes (e.g., difficulty of performing grip strength or long jump in very hot weather); to investigate this we categorized season as winter = December to January, spring = February to March, summer = April to June, monsoon = July to mid‐September, autumn = mid‐September to November (https://delhitourism.gov.in/delhitourism/aboutus/seasons_of_delhi.jsp).

### 2.5 Diet pattern generation

We investigated correlations among intakes of the different food groups to determine whether there was sufficient variability for pattern analyses; most correlations were very low although intakes of the different groups of fruits and vegetables were inter‐correlated as were intakes of organ plus red meats, poultry and fish, and eggs. PCA was conducted using varimax rotation to facilitate interpretation of the factor loadings. The number of components to retain for further analyses was based on both those with eigenvalues >1 and on where there was a break in the scree plot. The components were named according to which food groups had the greatest factor loadings, that is, those with values of loading >0.2. Principal component scores were then divided into terciles to indicate how closely each child's diet adhered to this component.

### 2.6 Outcomes investigated for their associations with dietary patterns

The outcomes of interest were those related to longer term poor health. HbA1c, a risk factor for diabetes, was used both as a continuous variable in analyses with diet patterns and as the proportion high HbA1c, defined as >5.43% which was the 90th percentile for American children aged 10–14 years (Saaddine et al., 2002). Low grip strength has been associated with poor health outcomes or mortality in several adult populations (Celis‐Morales et al., 2018; Leong et al., 2015), although associations in children are not clear. We used long jump as an indicator of lower limb strength to be similar to grip strength for upper limbs and cheaper and more suitable for field use than jumping mechanography (Ward et al., 2010).

We analyzed these outcomes and potential factors associated with diet with terciles of PCA components using linear regression for continuous outcomes and logistic regression for categorical variables. We first investigated how the independent sociodemographic variables and season were associated with particular diet patterns as outcomes. We then compared dietary patterns as exposures with anthropometric variables and body composition (FMI and FFMI) as outcomes since these variables may lie on the causal pathway between diet and NCD outcomes. Finally, we analyzed dietary patterns as exposures with risk factors for later chronic diseases (HbA1c, grip strength, and long jump) as outcomes. Age and sex were controlled for in the first analyses of risk factors for later disease because they are associated with all these outcomes. Subsequent multivariable analysis also controlled for sociodemographic factors found associated with diet patterns. We checked variance inflation factors to determine if there was multicollinearity.

## 3 RESULTS

**FIGURE 1:**
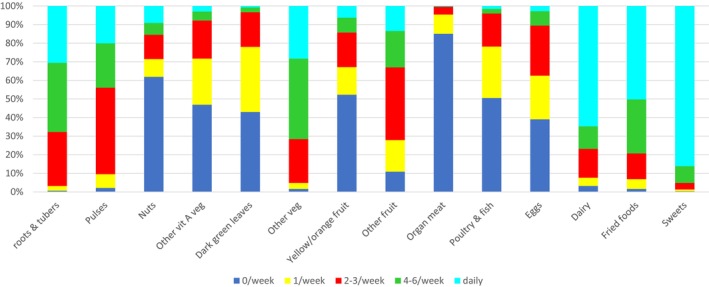
Frequency of intake of food groups.

The study included 742 children, 665 from the DIVIDS low‐birth weight cohort and 77 normal birth weight controls. For all children combined, the percentages of eating from each of the 14 food groups at each of the five intake frequencies are shown in Figure 1. The frequency of consumption of nuts, vitamin A‐rich fruits and vegetables, organ plus red meat, poultry and fish, or eggs was low, whereas consumption of other fruits and vegetables, dairy, fried foods, and sweets was frequent.

In the PCA, six components had eigenvalues >1. Although the scree plot (Figure S1) had no clear break in its slope, there was a slight elbow after the third principal component so we chose to use the first three components which, together, explained 36% of the diet variability. The Kaiser–Meyer–Olkin measure was 0.59, indicating sampling adequacy.

Table 1 shows factor loadings for food groups with bold numbers for those where the loading was >0.2. Based on the foods which contributed to the component, we called the first component ‘fruits and vegetables’ since all these food groups contributed, as did dairy and sweets. The second component was called ‘protein’ since the main contributing factors were meats, fish, and eggs, and the third component was called ‘sugar and fat’ since these were major contributors, whereas intakes of nonvitamin A‐rich fruits and vegetables and nuts had strong negative factor loadings. Using chi‐square to determine associations among terciles of adherence to each dietary pattern, we found that children with high adherence to the fruits and vegetables pattern had lower adherence to the sugar and fat pattern and vice versa, whereas the degree of adherence to the protein pattern was not associated with adherence to the other patterns (data not shown).

**TABLE 1:** Factor loadings for food groups contributing to the first three components in the principal component analysis.^a^

	Component 1	Component 2	Component 3
	Fruits and vegetables	Protein	Sugar and fat
Roots and tubers	**0.17**	−0.02	**0.37**
Pulses	**0.25**	−0.07	**0.23**
Nuts	0.18	0.09	**−0.42**
Vitamin A‐rich vegetables	**0.43**	−0.06	−0.07
Dark green leafy vegetables	**0.21**	0.07	**−0.32**
Other vegetables	**0.40**	−0.10	0.12
Yellow and orange fruits	**0.50**	−0.03	0.10
Other fruits	**0.39**	0.14	**−0.25**
Organ and red meats	−0.05	**0.51**	0.04
Poultry and fish	−0.01	**0.62**	0.07
Eggs	0.01	**0.51**	−0.07
Dairy	**0.22**	0.09	−0.16
Fried foods	0.03	0.16	**0.51**
Sweets	**0.21**	0.10	**0.38**
Explained variation in food groups	13.2%	12.6%	10.0%

^a^ Factor loadings from rotated components in the analysis; those with loadings greater than the absolute value of 0.2 are in bold and were used to identify the component.

Table 2 describes children's sociodemography, and Table 3 shows the anthropometry and body composition results according to their degree of adherence to each of the dietary patterns determined by PCA. Older children adhered more to the fruit and vegetable pattern, whereas Hindus and children from higher SES families or whose mothers had more education adhered less. Children who adhered more to this pattern had lower HAZ but there were no other associations between this diet and anthropometry or body composition. Girls and Hindus adhered more to the protein diet pattern and the middle SES tercile adhered least. Anthropometry and body composition were not significantly associated with this pattern. There was a tendency for older children and those from families with higher SES and maternal education to adhere more to the sugar and fat pattern. Adherence to this pattern was associated with higher height, but not HAZ, high indicators of width and body fat (BMI, waist, hip and mid‐upper‐arm circumferences, triceps and subscapular skinfolds, FMI), and no difference in FFMI. Adherence to all diet patterns varied with season of data collection. No diet pattern was associated with whether the child came from the original DIVIDS low‐birth weight cohort or the more recently recruited normal birth weight group.

**TABLE 2:** Participant characteristics according to their adherence to the three dietary patterns determined by principal component analysis.^a^ ^,^ ^b^ ^,^ ^c^

	Fruits and vegetables				Protein				Sugar and fat			
	Low	Medium	High	*p*	Low	Medium	High	*p*	Low	Medium	High	*p*
*N*	248	247	247		248	247	247		248	247	247	
Females	134 (54%)	134 (53%)	118 (48%)	.26	116 (47%)	123 (50%)	147 (60%)	.01	118 (48%)	133 (54%)	135 (55%)	.23
Age (years)	11.0 (1.0)	11.2 (1.0)	11.3 (1.0)	.004	11.2 (1.0)	11.2 (1.0)	11.1 (1.0)	.71	11.1 (1.0)	11.1 (0.9)	11.3 (1.1)	.08
Religion												
Hindu	223 (94%)	218 (88%)	203 (82%)	.001	199 (80%)	217 (87%)	238 (96%)	<.001	214 (86%)	213 (86%)	227 (92%)	.15
Muslim	13 (5%)	25 (10%)	34 (14%)		43 (17%)	23 (9%)	6 (2%)		27 (11%)	30 (12%)	15 (6%)	
Other	2 (1%)	4 (2%)	10 (4%)		6 (2%)	7 (3%)	3 (1%)		7 (3%)	4 (2%)	5 (2%)	
Mother's education												
None	38 (15%)	30 (12%)	42 (17%)	.001	42 (17%)	36 (15%)	32 (13%)	.15	38 (15%)	42 (17%)	30 (12%)	.01
Primary	27 (11%)	32 (13%)	37 (15%)		37 (15%)	25 (10%)	34 (14%)		42 (17%)	27 (11%)	27 (11%)	
Middle	52 (22%)	59 (24%)	84 (34%)		67 (27%)	79 (29%)	58 (23%)		62 (25%)	70 (28%)	63 (26%)	
Secondary	96 (39%)	94 (38%)	69 (28%)		84 (34%)	88 (36%)	87 (35%)		89 (36%)	86 (35%)	84 (34%)	
College	35 (14%)	30 (12%)	14 (6%)		17 (7%)	26 (11%)	36 (15%)		17 (7%)	21 (9%)	41 (17%)	
Mother's occupation												
Housewife	202 (81%)	204 (83%)	199 (81%)	.93	198 (80%)	208 (85%)	199 (81%)	.33	205 (83%)	205 (83%)	195 (80%)	.54
Employed	45 (18%)	40 (16%)	45 (18%)		48 (19%)	37 (15%)	45 (18%)		42 (17%)	39 (16%)	49 (20%)	
Student/other	1 (0.4%)	2 (0.8%)	2 (0.8%)		2 (0.8%)	0 (0%)	3 (1%)		1 (0.4%)	3 (1%)	1 (0.4%)	
Socioeconomic tercile												
Low	63 (25%)	77 (31%)	108 (44%)	<.001	73 (29%)	69 (28%)	106 (43%)	<.001	99 (40%)	90 (36%)	59 (24%)	<.001
Medium	88 (35%)	87 (35%)	72 (29%)		100 (40%)	84 (34%)	63 (26%)		87 (35%)	85 (34%)	75 (30%)	
High	97 (39%)	83 (34%)	67 (27%)		75 (30%)	94 (38%)	78 (32%)		62 (25%)	72 (29%)	113 (46%)	
Season												
Winter	29 (12%)	51 (21%)	48 (19%)	<.001	46 (19%)	53 (21%)	29 (12%)	.002	17 (7%)	44 (18%)	67 (27%)	<.001
Spring	50 (20%)	50 (20%)	55 (22%)		54 (22%)	45 (18%)	56 (23%)		40 (16%)	52 (21%)	63 (26%)	
Summer	97 (39%)	52 (21%)	40 (16%)		59 (24%)	58 (23%)	72 (29%)		72 (29%)	56 (23%)	61 (25%)	
Monsoon	52 (21%)	42 (17%)	37 (15%)		32 (13%)	42 (17%)	57 (23%)		71 (29%)	42 (17%)	18 (7%)	
Autumn	20 (8%)	52 (21%)	67 (27%)		57 (23%)	49 (20%)	33 (13%)		48 (19%)	53 (21%)	38 (15%)	
Birth weight												
<2.5 kg	219 (88%)	223 (90%)	223 (90%)	.71	220 (88%)	222 (90%)	223 (90%)	.84	228 (92%)	223 (90%)	214 (87%)	.14

^a^ Numbers are mean (SD) for age or # (%) for other variables.

^b^ Columns indicate terciles of low, medium, or high degree of adherence to the particular dietary pattern.

^c^ *p* values are from a linear regression for age and from chi‐square test for other variables.

**TABLE 3:** Participant anthropometry and body composition according to their adherence to the three dietary patterns determined by principal component analysis.^a^ ^,^ ^b^ ^,^ ^c^

	Fruits and vegetables				Protein				Sugar and fat			
	Low	Medium	High	*p*	Low	Medium	High	*p*	Low	Medium	High	*p*
*N*	248	247	247		248	247	247		248	247	247	
Height (cm)	138 (8.8)	139 (9.4)	138 (9.0)	.86	139 (8.9)	139 (9.1)	138 (9.2)	.56	137 (8.4)	138 (9.1)	139 (9.6)	.02
Height‐for‐age Z	−0.87 (0.96)	−1.04 (1.03)	−1.11 (1.01)	.02	−0.99 (1.03)	−0.98 (0.99)	−1.06 (1.00)	.67	−1.12 (0.96)	−0.94 (1.05)	−0.97 (1.00)	.11
BMI (kg/m^2^)^d^	15.3 (14.1, 17.6)	15.2 (13.9, 17.4)	15.1 (13.9, 17.1)	.55	15.2 (14.0, 17.4)	15.1 (13.9, 17.3)	15.2 (13.9, 17.2)	.87	15.0 (13.8, 17.0)	14.9 (13.8, 16.6)	15.6 (14.2, 18.1)	.006
BMI‐for‐age Z	−0.87 (1.39)	−1.03 (1.48)	−1.14 (1.49)	.13	−0.97 (1.46)	−1.08 (1.50)	−1.00 (1.41)	.70	−1.08 (1.48)	−1.17 (1.45)	−0.80 (1.42)	.01
Waist circumference (cm) ^¶^	55.4 (52.0, 62.4)	55.0 (51.4, 61.5)	55.2 (51.3, 60.7)	.62	55.3 (52.1, 61.3)	55.5 (51.0, 61.3)	55.0 (51.2, 61.9)	.68	54.9 (51.5, 60.5)	54.9 (51.5, 60.3)	56.5 (52.4, 63.1)	.007
Hip circumference (cm)	69.3 (8.3)	69.5 (9.1)	68.9 (8.5)	.75	69.2 (8.7)	69.3 (8.7)	69.1 (8.6)	.98	68.3 (8.0)	68.7 (8.9)	70.7 (9.0)	.006
MUAC	19.3 (2.8)	19.3 (3.2)	19.0 (2.9)	.44	19.3 (3.1)	19.2 (3.0)	19.2 (2.9)	.88	19.0 (3.0)	19.0 (2.9)	19.7 (3.1)	.007
Triceps skinfold (mm) ^¶^	8.6 (6.4, 13.4)	8.4 (6.2, 13.2)	8.2 (6.4, 12.4)	.73	8.4 (6.4, 12.3)	8.4 (6.4, 13.2)	8.4 (6.4, 13.8)	.98	8.1 (6.4, 12.0)	8.0 (6.2, 12.0)	9.4 (6.6, 14.4)	.005
Subscapular skinfold (mm) ^¶^	7.4 (5.8, 11.8)	7.8 (5.8, 13.6)	7.8 (6.0, 12.8)	.39	7.6 (6.0, 12.5)	8.0 (5.8, 13.0)	7.8 (5.8, 12.8)	.96	7.2 (5.8, 10.7)	7.4 (5.6, 12.6)	8.6 (6.4, 14.2)	.008
FMI (kg/m^2^) ^¶^	1.7 (1.1, 3.5)	1.8 (1.0, 3.4)	1.7 (0.9, 3.2)	.36	1.7 (1.1, 3.2)	1.7 (0.9, 3.4)	1.8 (1.1, 3.4)	.65	1.5 (1.0, 3.0)	1.7 (1.0, 2.9	2.0 (1.2, 4.1)	.002
FFMI (kg/m^2^)	13.2 (1.0)	13.1 (1.1)	13.1 (1.1)	.54	13.2 (1.1)	13.1 (1.0)	13.1 (1.0)	.76	13.2 (1.1)	13.0 (1.0)	13.2 (1.0)	.15

Abbreviations: BMI, body mass index; FFMI, fat‐free mass index; FMI, fat mass index; MUAC, mid‐upper‐arm circumference.

^a^ Numbers are mean (SD) or median (25th and 75th percentiles).

^b^ Columns indicate terciles of low, medium, or high degree of adherence to the particular dietary pattern.

^c^ *p* values are from linear regressions; although not shown, in all cases where *p* < 0.05, it was the highest diet adherence which was significantly different from the lowest which was the reference.

^d^ Data were log‐transformed to normalize distributions so values are median (25th and 75th percentiles and *p* values are from regressions using log values.

In analyses adjusted for age and sex, higher adherence to the fruits and vegetables pattern was associated with lower grip strength, shorter long jump, lower HbA1c, and lower odds of high HbA1c (Table 4). Additional adjustment for religion, socioeconomic status, maternal education, and season, i.e., variables associated with dietary patterns, resulted in little change to these associations. In the multivariable analyses, all variance inflation factors were <3, indicating no problem of multicollinearity. There were no significant associations either in analyses controlled for age and sex or in multivariable analyses between the protein or sugar and fat diet patterns and these markers of risk of later disease.

**TABLE 4:** Association of risk factors for chronic disease with adherence to the three dietary patterns determined by principal component analysis.^a^

	PCA tercile	Mean (SD) or # (%)	Regression controlling for age and sex		Multivariable regression^b^	
			Coefficient (95% CI)	*p*	Coefficient (95% CI)	*p*
Fruits and vegetables						
Grip strength (kg)	Low	12.8 (3.1)	Reference		Reference	
	Medium	12.9 (3.4)	−0.27 (−0.75, 0.20)	.26	−0.45 (−0.94, 0.03)	.07
	High	12.7 (3.0)	−0.52 (−1.00, −0.05)	.03	−0.65 (−1.15, −0.14)	.01
Long jump (m)	Low	1.31 (0.23)	Reference		Reference	
	Medium	1.29 (0.22)	−0.03 (−0.07, 0.01)	.10	−0.03 (−0.07, 0.01)	.09
	High	1.28 (0.25)	−0.05 (−0.09, −0.01)	.02	−0.06 (−0.10, −0.02)	.004
HbA1c (%)	Low	5.5 (0.3)	Reference		Reference	
	Medium	5.3 (0.3)	−0.11 (−0.17, −0.05)	<.001	−0.08 (−0.14, −0.02)	.007
	High	5.3 (0.4)	−0.14 (−0.20, −0.07)	<.001	−0.10 (−0.16, −0.03)	.002
HbA1c > 5.43%	Low	129 (52%)	Reference^b^		Reference^b^	
	Medium	97 (39%)	0.62 (0.43, 0.89)	.01	0.74 (0.50, 1.09)	.13
	High	82 (33%)	0.47 (0.33, 0.68)	<.001	0.56 (0.37, 0.84)	.006
Protein						
Grip strength (kg)	Low	12.9 (3.3)	Reference		Reference	
	Medium	12.8 (3.1)	−0.02 (−0.49, 0.46)	.94	−0.06 (−0.54, 0.41)	.80
	High	12.7 (3.0)	0.03 (−0.45, 0.50)	.92	0.13 (−0.37, 0.63)	.61
Long jump (m)	Low	1.31 (0.24)	Reference		Reference	
	Medium	1.29 (0.23)	−0.02 (−0.05, 0.02)	.43	−0.01 (−0.05, 0.03)	.64
	High	1.28 (0.23)	−0.01 (−0.05, 0.03)	.76	−0.01 (−0.05, 0.03)	.58
HbA1c (%)	Low	5.4 (0.4)	Reference^b^		Reference	
	Medium	5.4 (0.3)	−0.03 (−0.09, 0.03)	.39	−0.02 (−0.08, 0.04)	.49
	High	5.4 (0.4)	−0.04 (−0.10, 0.02)	.24	−0.05 (−0.11, 0.01)	.10
HbA1c > 5.43%	Low	110 (44%)	Reference		Reference^b^	
	Medium	99 (40%)	0.84 (0.59, 1.20)	.34	0.82 (0.56, 1.21)	.32
	High	99 (40%)	0.84 (0.59, 1.21)	.35	0.74 (0.49, 1.11)	.14
Sugar and fat						
Grip strength (kg)	Low	12.7 (3.1)	Reference		Reference	
	Medium	12.6 (3.1)	−0.03 (−0.51, 0.44)	.89	−0.16 (−0.64, 0.31)	.50
	High	13.1 (3.3)	0.26 (−0.22, 0.73)	.29	0.10 (−0.40, 0.61)	.69
Long jump (m)	Low	1.31 (0.23)	Reference		Reference	
	Medium	1.28 (0.23)	−0.02 (−0.06, 0.01)	.22	−0.01 (−0.05, 0.03)	.48
	High	1.29 (0.24)	−0.02 (−0.06, 0.02)	.39	0.01 (−0.03, 0.05)	.53
HbA1c (%)	Low	5.4 (0.4)	Reference		Reference	
	Medium	5.4 (0.4)	−0.02 (−0.08, 0.04)	.48	0.00 (−0.06, 0.06)	.91
	High	5.4 (0.3)	0.00 (−0.06, 0.06)	.95	0.03 (−0.04, 0.09)	.42
HbA1c > 5.43%	Low	107 (43%)	Reference^b^		Reference^b^	
	Medium	94 (38%)	0.82 (0.57, 1.18)	.29	0.99 (0.67, 1.46)	.95
	High	107 (43%)	1.07 (0.74, 1.53)	.73	1.34 (0.89, 2.01)	.16

^a^ Analyses were by linear regression for continuous variables and logistic regression for HbA1c. The table presents coefficients (95% confidence intervals) from linear regression or odds ratios (95% confidence intervals) from logistic regression. The first set of analyses was controlled for age and sex and the multivariable analyses for age, sex, religion, socioeconomic tercile, mother's education, and season of data collection.

^b^ Sample sizes are reduced in multivariable analyses due to missing data; *N* = 738 for grip strength, 731 for long jump, and 723 for HbA1c.

## 4 DISCUSSION

Three dietary patterns were derived from PCA, two of which, the fruits and vegetables pattern and the fat and sugar pattern, correspond, respectively, to healthy and unhealthy diets found in other studies of children (Shen et al., 2022; Veldheer et al., 2021). The protein pattern of flesh food consumption was seen in a small proportion of this cohort. As seen by others (Huybrechts et al., 2017; Sauvageot et al., 2016), the healthy fruits and vegetables pattern was associated with lower HbA1c, and the unhealthy fat and sugar pattern was associated with higher indicators of body fat, although not with the NCD risk factor outcomes.

We focused on the associations of diet patterns with outcomes, e.g., grip strength and HbA1c, which have shown consistent associations with NCDs in many populations (Leong et al., 2015; Saaddine et al., 2002) rather than on the anthropometric and body composition variables which are on the causal pathway to NCDs. In a low‐income population such as DIVIDS, with generally low values of anthropometric variables, as indicated by the negative HAZ and BMIZ values, it remains unclear whether increasing child size is advantageous or not. It would be difficult to persuade parents not to give foods high in fat and sugar on the basis of increased fat deposition when their children are generally small and many were born with low birth weight. However, the skewed results for FMI and anthropometric variables associated with body fat suggest that a subset of these children may be at risk for later NCDs even though at this relatively young age the children may have not yet had time to develop NCDs which usually manifest later in life. Thus, it is concerning that we found a very high prevalence of high HbA1c, though we recognize we used a cut‐off designed for a very different population.

The diet pattern which was associated with lower HbA1c was the fruits and vegetable pattern which was eaten more by children of low SES and with mothers of lower education. Higher adherence to the fruits and vegetables pattern was associated with shorter jumps; this may be because the pattern was also associated with lower HAZ but we did not control for anthropometric variables in analyses of NCD outcomes because we speculated they were on the causal pathway.

Diet patterns have different associations with sociodemographic variables in different countries with data from high‐income countries generally showing that children from families with greater wealth or parental education adhere more to healthy patterns and less to unhealthy patterns, whereas the evidence from low‐ and middle‐income countries is less clear (Hinnig et al., 2018). We found that children from families of higher socioeconomic status or with more educated mothers had lower adherence to the healthy fruits and vegetables pattern. We are aware of two other studies of dietary patterns of Indian children and adolescents which found somewhat different results to ours. The ISCOLE study of similar‐aged children to ours, found in India, as well as in 11 other countries of a wide range of income, higher socioeconomic status and education were associated with lower odds of consuming an unhealthy diet and greater odds of consuming a healthy diet since it is expected that well‐educated parents are more aware about concerns related to nutrition and health (Manyanga et al., 2017). The authors themselves were surprised at the consistency of this association across socioeconomic levels and speculated that, in the populations they studied, the nutrition transition was almost complete which is why all countries showed the same socioeconomic gradients with diet. A study in Mysore found a ‘snack and fruit’ diet pattern which was similar to our fruits and vegetables pattern; similar to our study, families of unskilled workers were more likely to adhere to this pattern (Kehoe et al., 2014), indicating that this was possibly a social or cultural trend specific to India. This trend continues over time despite changes in diet and epidemiological transition and thus this study could be important from a policy perspective, especially since there is limited information on dietary patterns in India. They also found that Muslims adhered more to the ‘snack and fruit’ pattern, whereas Hindus adhered more to a ‘lacto‐vegetarian’ pattern which was not similar to the patterns we found. Contrary to our expectations, in our cohort, Hindus had higher adherence to the protein pattern and lower adherence to the fruits and vegetables pattern. This may be a result of the particular neighborhood from which the DIVIDS children were recruited for the original trial of vitamin D (Trilok‐Kumar et al., 2011). Muslim families in the area were generally of lower socioeconomic status than the Hindu families so may have eaten cheaper vegetables rather than much meat. Comparing the results of these studies of similarly aged Indian children indicate how local specific diet patterns can be so that, for interventions to improve children's diets, preliminary research needs to be conducted to determine locally important foods to consider.

Strengths of the study were its large, well‐characterized cohort of children from lower income Indian families and its multiple anthropometric, body composition, and health risk factor measurements. Study limitations include the possibly inappropriate cut‐off for high HbA1c, the lack of information on quantity of intake from an FFQ, and the low number of control children which precluded investigation of whether low birth weight modified the diet–NCD risk factor associations. The difficulty of recruiting control children, who, to be eligible, needed families to still have their birth record cards, likely resulted in a bias toward families with more stable housing and enough space to store records for many years. The unavailability of documented birth proof of NBW children as required by the inclusion criteria resulted in a small number of controls. The low‐birth weight group may have been similarly biased toward more stable families since the children lost over time from the original DIVIDS study were from families of lower socioeconomic status (Trilok‐Kumar et al., 2015). These biases reduce our ability to extrapolate the findings.

In conclusion, we have determined dietary patterns in a group of low‐income Indian children, most of whom were born with low birth weight and are, thus, at higher risk of NCDs. Our results are similar to one Indian study available showing that high intake of fruits and vegetables reduces the risk of diabetes, as indicated by lower HbA1c. With all Indian surveys showing increasing trends of NCDs, the importance of eating fruits and vegetables regularly is a simple message related to foods affordable even by low‐income families in India which can be communicated to the population and translated into policies and programs.

## AUTHOR CONTRIBUTIONS

**Geeta Trilok‐Kumar:** Conceptualization (equal); funding acquisition (equal); project administration (equal); resources (equal); supervision (equal); writing – original draft (equal). **Anku Malik:** Data curation (equal); investigation (equal); methodology (equal); project administration (equal); writing – review and editing (equal). **Yamini Gusain:** Data curation (equal); investigation (equal); methodology (equal); project administration (equal); writing – review and editing (equal). **Eve Millerot:** Data curation (equal); formal analysis (equal); software (equal); writing – original draft (supporting). **Renuka Pathak:** Project administration (supporting); supervision (equal); writing – review and editing (equal). **Suzanne Filteau:** Conceptualization (equal); formal analysis (equal); funding acquisition (supporting); software (equal); visualization (equal); writing – original draft (equal).

## FUNDING INFORMATION

The DIVIDS‐3 follow‐up of the cohort was funded by the Department of Biotechnology (DBT) and Wellcome Trust‐DBT India‐Alliance # IA/CPHS/17/1/503334. SF's time was funded, in part, by the UK Medical Research Council, grant MR/V000578/1.

## CONFLICT OF INTEREST STATEMENT

The authors declare no conflict of interest.

## ETHICS STATEMENT

The study conforms to the Declaration of Helsinki; US Federal Policy for the protection of human subjects. The study was approved by the Institutional Ethics Committee of Institute of Home Economics (University of Delhi) (Ethical Clearance Number‐IHE/2018/1146).

## Supporting information


Figure S1

## Data Availability

The data that support the findings of this study are available on request from the corresponding author. The data `are not publicly available due to privacy or ethical restrictions.
