# Supplementary material for: Dietary patterns of Indian school‐aged children and associations with markers of chronic disease risk
Source: Food Sci Nutr. 2023 Aug 16;11(11):7070–9. doi: 10.1002/fsn3.3631 (PMC10630838; doi:10.1002/fsn3.3631)
Supplement: Supplementary file 1 — Figure S1 [file FSN3-11-7070-s001.docx]

**Supplementary Figure 1. Scree plot of eigenvalues from the principal component analysis**

**of dietary patterns**
